# Vitamin C intake and risk of renal cell carcinoma: a meta-analysis

**DOI:** 10.1038/srep17921

**Published:** 2015-12-08

**Authors:** Li Jia, Qingling Jia, Yonggang Shang, Xingyou Dong, Longkun Li

**Affiliations:** 1 Department of urology, Xinqiao Hospital, Third Military University, Chongqing, 40037, China; 2 Department of Nephrology, Xinqiao Hospital, Third Military University, Chongqing, 40037, China

## Abstract

Studies have showed that vitamin C intake is linked to renal cell carcinoma risk, however, the results were inconsistent. Hence, the present meta-analysis was to examine the association between vitamin C intake and RCC risk. We searched the published studies that reported the relationship between vitamin C intake and RCC risk using PubMed and Embase up to January 2015. Based on a fixed effects model, RR and the corresponding 95% CI were used to assess the pooled risk. 3 prospective cohort studies and 7 case-control studies were included. The overall RR (95% CI) of RCC for the highest vs. the lowest levels of vitamin C intake was 0.78(0.69,0.87). Little evidence of heterogeneity was found. In the subgroup analyses, we found an inverse association between vitamin C intake and RCC risk in the case-control studies but not in the prospective cohort studies. Additionally, this association between vitamin C intake and RCC risk was not differed by population distribution. Our study provides evidence that vitamin C intake is associated with a reduced RCC risk. However, our conclusion was just based on ten including studies, so more high-quality of case-control studies or cohort studies which report this topic are needed.

Cancer is a major public health problem in the world including the United States. One in four deaths in the United States is due to cancer^1^. The kidney is an essential organ that maintains the homeostatic balance of human body. However, kidney cancer remains the sixth commonest cause of cancer among men and the eighth commonest among women in the United States^2^. Adult kidney cancer that arises in the renal parenchyma are mainly adenocarcinomas, also known as renal cell carcinoma(RCC). The incidence of kidney cancer has reached a plateau, with cancer generally diagnosed at earlier stages in the past 3 decades^3^. In 2014, it is estimated that there will be 63,920 new cases of kidney cancer in the United States, with 13,860 estimated deaths from this disease and an overall 5-year survival rate of approximately 70%^1^.

Age, race, gender, smoking and obesity have been established risk factors for RCC^4^. Dietary factors also appear to play a role, with higher fruit and vegetable intake linked to lower risk of this disease in several studies^2^,^5^,^6^,^7^,^8^,^9^,^10^,^11^,^12^,^13^,^14^. Fruit and vegetables are rich in anti-oxidants and other phytochemicals, which have been proposed to prevent several diseases^15^, including cancers^16^.

As one of free-radical scavenging antioxidant nutrients, vitamins C is capable of inhibiting oxidative DNA damage, mutagenesis, and tumor growth^17^,^18^. Studies indicated that vitamin C intake might be associated with a decreased risk of several types of cancer (cervical and colon)^19^,^20^. Are there others? I do believe so and there are some in which vitamin C intake increased risk such as there was an increasing breast cancer rate with increasing vitamin C intake^21^. However, the relationship of vitamin C intake and RCC is unclear.

In 2006, a case-control study showed that there was an inverse association between vitamin C intake and RCC risk^7^. But the result was inconsistent with others. In 2012, another case-control study indicated that there was no association between vitamin C intake and RCC risk^12^, and a cohort study in 2014 showed the similar result. Hence, we conducted a meta-analysis of the relevant studies by combining the results from the published observational studies to assess the relationship between vitamin C intake and the risk of RCC. In addition, we conducted two subgroup analyses to assess the effects of various study characteristics on the relationship between dietary vitamin C intake and RCC risk.

## Material and Methods

### Literature search strategy

We generally followed the Meta-Analysis of Observational Studies in Epidemiology (MOOSE) guidelines^22^, we conducted a systematic literature search of the PubMed and Embase through January 1980 to June 2015 to identify published studies that reported the association of dietary vitamin C intake of RCC with following search terms: “dietary vitamin C intake”, “vitamin C intake” and “vitamin C” in combination with “renal cell cancer”, “kidney cancer” and “renal cell carcinoma”. Reference lists of the retrieved articles were also reviewed. If necessary, we contacted the authors of the original studies for the required data.

### Study selection

The studies that met the following criteria could be included: 1. a prospective cohort design or case-control design; 2. the exposure of interest was vitamin C intake and the kind of intake is only from foods or supplements; 3. the outcome was RCC incidence; 4. the RRs and the corresponding 95% CIs for the highest compared with the lowest levels of vitamin C intake were reported.

### Data extraction

For data extraction, the following information was extracted: the last name of the first author, publication year, characteristics of study population (age and sex), geographic region, follow-up length, number of cases or size of cohort; RR from the most fully adjusted model for the highest compared with the lowest levels of vitamin C intake and the corresponding 95% CI adjustment from the multivariable model; statistical adjustment for the potential confounding factors. Two authors independently performed the literature search, studies selection, and data extraction, any disagreements were resolved by discussion.

### Statistical analysis

STATA version 12.0 was used to conducted all analyses. Based on a fixed-effects model, RR and the corresponding 95% CI were used to measured the association between vitamin C intake and RCC risk. Homogeneity test was performed with the use of Q statistic at the P < 0.05 level of significance and I^2^ statistic, which is a quantitative measure of inconsistency across studies^23^. If the Q statistic at the P < 0.05 level of significance and I^2^ > 50%, the random effects model was choosed; however, If the Q statistic at the P > 0.05 level of significance and I^2^ < 50%, the fixed effects model was selected. Subgroup analyses were performed to identify the source of heterogeneity, if possible, and the effect of the potential factors on the overall risk estimate. Additionally, we conducted a sensitivity analysis to investigate the influence of a single on overall risk estimate by omitting one study in turn. Potential publication bias was assessed by Begg rank correlation test and Egger linear regression test^24^,^25^. All statistical tests were two-sided and P < 0.05 was considered statistically significant, except where otherwise specified.

## Results

### Literature search

We initially identified 908 potential articles from Pubmed (n = 147) and Embase (n = 761). Most were excluded because: 1. most studies from Embase were pharmaceutical research. 2. They were reviews or animal experiments. 3. They were not case-control or cohort studies. 4. The exposure was not vitamin C or the endpoint was not RCC. 5. The duplicated citations. Then 15 potentially eligible studies were left, by more detailed evaluation, another 5 studies were excluded. At last, 10 studies (3 prospective cohort studies comprising 259845 participants and 743 incident cases and 7 case-control studies involving 4439 cases and 10580 controls)^2^,^5^,^6^,^7^,^8^,^9^,^10^,^11^,^12^,^13^,^14^ were included.

### Study Characteristics

The main characteristics of the 10 studies are presented in Table 1. These studies were published from 1996 to 2014. Among them, one^8^ only included males and the other one^2^ only included females. 4 studies were based on Europeans (1 in Swedes, 1 in Italians, 1 in Germans and 1 in Danes, respectively), and 6 were based on the North-Americans(5 in Americans and 1 in Canadians, respectively). The ranges of age of cases and controls is 20–85 in the case-control studies, but the age of participants from 3 prospective cohort studies was in the rang of 40–79. The length of follow-up ranged from 15 to 20 years in the cohort studies. All the RRs in each study were based on the highest compared with the lowest levels of vitamin C intake. All studies used food-frequency questionnaires for assessment of vitamin C intake and adjusted for a wide range of potential confounders, including age, BMI, smoking, educational level, etc.

### Meta-analysis

**Figure 1:**
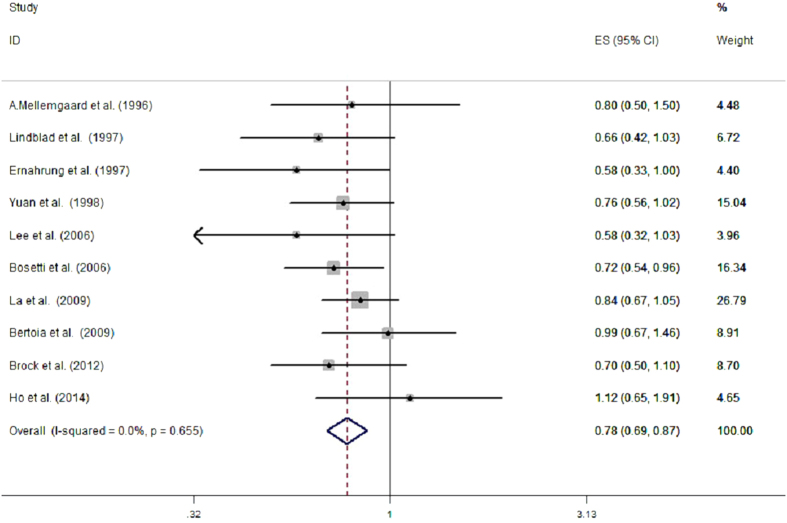
Meta-analysis of 10 studies that assess vitamin C intake and RCC risk.

10 epidemiological studies (7 case-control studies and 3 prospective cohort studies) reported the association between vitamin C intake and risk of renal cell cancer. The multivariable-adjusted RRs for each study and the combined RR for the highest compared with the lowest levels of vitamin C intake are presented in Fig. 1. The RRs from individual studies varied from 0.58 to 1.12. In the 10 included studies, one reported^6^ an inverse association between vitamin C intake and risk of RCC, however, others found null associations. Overall, there was a statistically significant inverse relationship between vitamin C intake and the risk of RCC, the pooled RR was 0.78 (95% CI, 0.69, 0.87). Little evidence of heterogeneity was found (P = 0.655, I^2^ = 0.0%).

**Figure 2:**
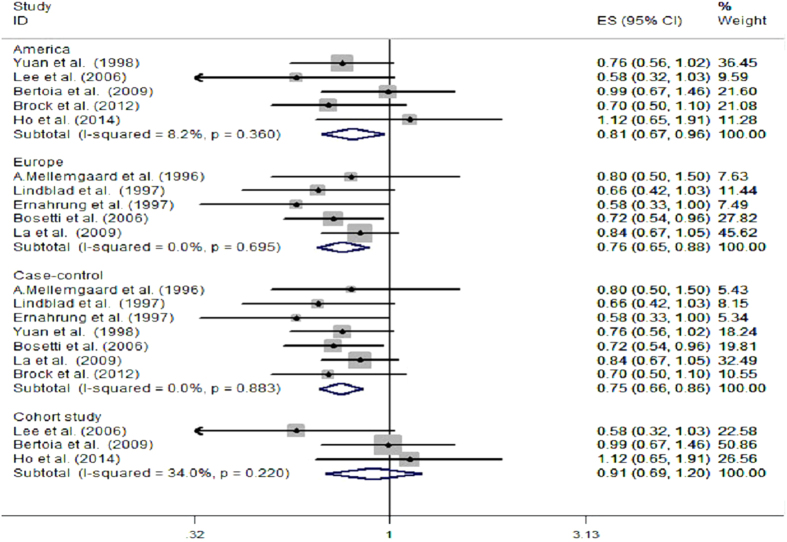
Subgroup analysis of vitamin C intake and RCC risk in different study designs and in different populations.

Fig. 2 present the results of subgroup analysis according to different populations and design patterns. There was little heterogeneity were observed within any subgroup-analyses. According to different populations, we found an inverse association between vitamin C intake and risk of RCC both in Americans and Europeans, the pooled RR and 95% CI was 0.81(0.67, 0.96), 0.76(0.65, 0.88), respectively. According to study design patterns, a significant inverse association between vitamin C intake and risk of RCC was observed in case-control studies, however, it showed that there was no statistically significant association in prospective cohort studies (the RR and 95% CI was 0.75(0.66, 0.86), 0.91(0.69, 1.20), respectively).

**Figure 3:**
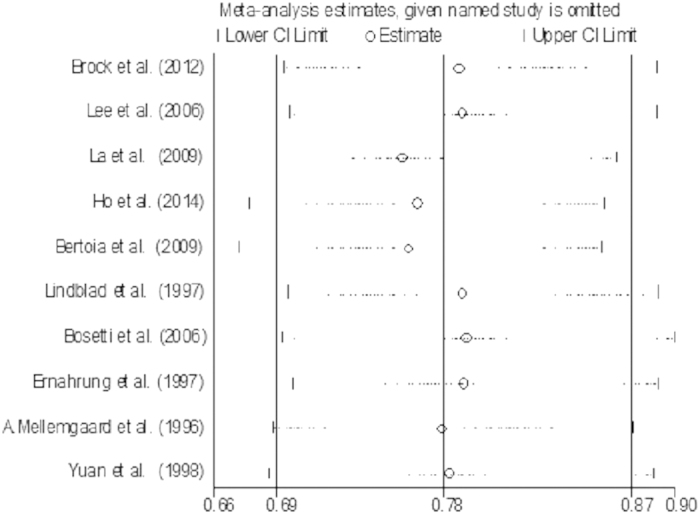
Funnel plot of sensitivity analysis for vitamin C intake and RCC risk.

Sensitivity analysis was conducted to assess the influence of a single study on the overall risk estimate by omitting one study in each turn. The combined RRs were all statistically significant and similar with one another, with a narrow range from 0.76 (95% CI: 0.66, 0.87) to 0.79 (95% CI: 0.69, 0.90), it revealed that the result was statistically stable and reliable. The result of sensitivity analysis is presented in Fig. 3.

**Figure 4:**
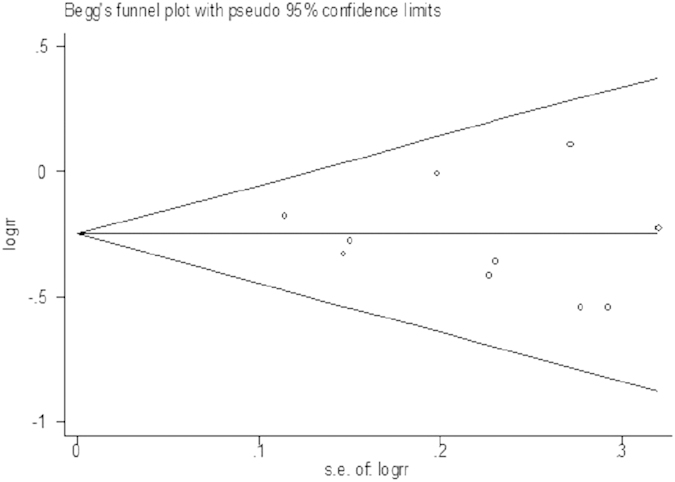
Funnel plot of publication bias for vitamin C intake and RCC risk.

Begg’s and Egger’s regression test showed a low probability of publication bias in our study (P = 0.515). The funnel plot of the studies is presented in Fig. 4.

## Discussion

The effect of antioxidants on the primary prevention of cancer has raised considerable interest in the recent years. Vitamin C which is one of the most common antioxidants plays an important role in our body metabolism. However, the epidemiological studies have reported inconsistent association of vitamin C intake with the risk of RCC. The present meta-analysis addresses this point by summarizing the updated evidence from published epidemiological studies and shows that there would exist an inverse association between vitamin C intake and risk of RCC, in other words, vitamin C intake may reduce the risk of RCC.

Heterogeneity is often a concern in a meta-analysis. However, little evidence of heterogeneity was observed across our study, which meant that there was little between-study variation. This could be partially explained by the following facts: all the studies were conducted in western countries, in where populations share much in terms of genetic background, lifestyle and dietary patterns; all the studies were published in English and had a high quality assessment, and the results from each original study was adjusted for a wide range of potential confounders, eliminating the most obvious confounding factors.

In the subgroup analyses, the pooled risk estimate of 7 case-control studies showed an inverse association between vitamin C intake and the risk of RCC, however, this significant inverse relationship was not found in the 3 prospective cohort studies. Although a higher level of statistical evidence was detected in the cohort studies than that in the case-control studies, we noted that these 3 prospective cohort studies with a small case sample size, and none had a case sample size of more than 300. In addition, the association was not differed by population distribution, the inverse association between vitamin C intake and risk of RCC was found both in North-Americans and Europeans. The subgroup analyses added additional evidence for the association between vitamin C intake and a risk of risk of RCC.

It has been hypothesized that the risk of RCC may be associated with compromised hemodynamics or metabolic disturbances as they relate to factors induced by hypoxia (hypoxia-inducible factors) and von Hippel-Lindau proteins and the presence of oxidative stress. As an antioxidant, the role of vitamin C intake has been focused on maintaining health and reducing the risk of noncommunicable diseases such as cancer. In 1990 s, lots of evidence showed that abundant consumption of fruits and vegetables including wealth vitamin C have been associated with a decreased risk of several chronic diseases including cardiovascular diseases^26^ and cancer^27^. In 1994, Okamoto *et al.* revealed that increased lipid peroxidation occurred in primary RCC as compared to surrounding cancer free tissues^28^. Byproducts of lipid per-oxidation have been shown to react with renal DNA to form adducts^29^,^30^, misrepair of the damage to DNA induced by these adducts can lead to mutations in proto-oncogenes and/or tumor suppressor genes, a critical step in the process of changing a normal cell to a malignant phenotype^31^.

Some limitations of our present study should be considered. First, the present findings were mainly based on case-control studies, although adjusted for adjusted for a wide range of potential confounders, the recall bias and selection bias were inevitable. Additionally, there were a small case sample size in the 3 prospective cohort studies, and they were based on elderly populations. Second, most of our included studies contained both male and female, however, two studies^2^,^8^ respectively included just only male or female. Female estrogen could inhibit renal cell carcinoma cell progression through estrogen receptor-β activation^32^. Due to the hormonal factors the two included studies my change the results of our study, so more adjusted for male or female respectively studies were needed. Third, our included studies were most based on European population, with different population distribution the race, lifestyle, dietary habit would be different, the relationship between vitamin C intake and RCC risk may be different, hence more global polycentric studies association between vitamin C intake and risk of RCC were needed. Finally, we could not completely rule out such bias because of the limited number of studies, although there was no evidence of publication bias.

## Conclusions

Our present meta-analysis provides evidence that vitamin C intake is associated with a reduced risk of RCC. But our conclusion was just based on ten including studies, so more high-quality of case-control studies or cohort studies which report the association between vitamin C intake and risk of RCC are needed.

## Additional Information

**How to cite this article**: Jia, L. *et al.* Vitamin C intake and risk of renal cell carcinoma: a meta-analysis. *Sci. Rep.* **5**, 17921; doi: 10.1038/srep17921 (2015).

## Figures and Tables

**Table 1:** The characteristics of the included studies.

Study	>Year	Population	Design	Sex	Age	Sample Size(n)	Questionnaire	Intake	Exposure range	Adjusted RR	Variables used in multivariate model
Brock *et al.*	2012	USA	Case-control	female/male	40–85	323/1827	Mailed Questionnaire	Mg/d Total	Q5>112,Q1<53	0.70(0.50,1.10)	Age, sex, proxy status, years of smoking, number Of cigarettes smoked per/d,never/ever smoke, BMI age 40 years, blood pressure, alcohol consumption, fat consump- tion and energy
Lee *et al.*	**2006**	USA	Cohort study (prospective)	female/male	40–75	248/136587	FFQ	Total	Q5:-,Q1:-	0.58(0.32,1.03)	BMI, history of hypertension, parity, history of diabees, smoking status,multivitamin use, alcohol intake, and total energy intake.
La *et al.*	2009	Canada	Case-control	female/male	20–76	1138/5039	FFQ	Total	Q5:195,Q1:-	0.84(0.67,1.05)	Sex, 10-year age group, province, total fat body mass index, alcohol drinking, pack-year smoking, processed meat, saturated fat, monounsaturate fat, transfat, cholesterol, and total energy intake.
Ho *et al.*	2014	USA	Cohort study (prospective)	female	50–79	240/96196	FFQ	Total Food Supplement	Q5>585,Q1<96.3 Q5>135.3,Q1<61.2 Q5>560,Q1:0(mcg)	1.12(0.65,1.91) 1.02(0.66,1.58) 1.17(0.66,2.05)	Micronutrients, age, clinical trial, race, education, BMI, hypertension, smoking status, oral contraceptive use, hysterectomy ever, oophorectomy ever, physical activity, and energy intake.
Bertoia *et al.* 2009	2009	USA	Cohort study (prospective)	male	50–69	255/27062	FFQ	Total	Q5:161,Q1:50	0.99(0.67,1.46)	Age, BMI, education level, measured systolic and diastolic blood pressure, self-reported history of hypertension, leisure-time physical activity, years of smoking, total number of cigarettes perday, trial intervention group and alcohol consumption, total energy intake, serum cholesterol.
Lindblad *et al.*	1997	Sweden	Case-control	female/mal	20–79	379/350	Self-administered questionnaire	Total Food	Q5:123,Q1<95 Q5:108.7,Q1<48.9	0.66(0.42,1.03) 0.83(0.53,1.31)	Age, sex, BMI, cigarette smoking, educational level
Bosetti *et al.*	2006	Italy	Case-control	female/male	24–79	767/1534	FFQ	Total	Q5:-,Q1:89.41	0.72(0.54,0.96)	Sex, study centre, period of interview, age, education, BMI, smoking habit, alcohol drinking and family history of kidney cancer
Ernahrung *et al.*	1997	Germany	Case-control	female/male	20–75	277/286	FFQ	Total	Q5:-,Q1:-	0.58(0.33,1.00)	Age, gender, educational status, tobacco moking and alcohol consumption.
A. *et al.*	1996	Denmark	Case-control	female/male	20–79	351/340	FFQ	Total	Q5>102,Q1<42	0.80(0.50,1.50)	Age, total energy intake, smoking, BMI,and socio-economic.
Yuan *et al.*	1998	USA	Case-control	female/male	25–74	1204/1204	FFQ	Total	Q5:-,Q6:-	0.76(0.56,1.02)	Level of education, BMI, history Of hypertension, number of cigarettes per day, current smoking status, total grams of analgesics consumed over lifetime and regular use of amphetamines
